# Quantifying the propagation of distress and mental disorders in social networks

**DOI:** 10.1038/s41598-018-23260-2

**Published:** 2018-03-22

**Authors:** Marialisa Scatà, Alessandro Di Stefano, Aurelio La Corte, Pietro Liò

**Affiliations:** 1 University of Catania, Dipartimento di Ingegneria Elettrica, Elettronica e Informatica, Catania, CNIT 95125 Italy; 2 University of Cambridge, Computer Laboratory, Cambridge, CB3 0FD UK

## Abstract

Heterogeneity of human beings leads to think and react differently to social phenomena. Awareness and homophily drive people to weigh interactions in social multiplex networks, influencing a potential contagion effect. To quantify the impact of heterogeneity on spreading dynamics, we propose a model of coevolution of social contagion and awareness, through the introduction of statistical estimators, in a weighted multiplex network. Multiplexity of networked individuals may trigger propagation enough to produce effects among vulnerable subjects experiencing distress, mental disorder, which represent some of the strongest predictors of suicidal behaviours. The exposure to suicide is emotionally harmful, since talking about it may give support or inadvertently promote it. To disclose the complex effect of the overlapping awareness on suicidal ideation spreading among disordered people, we also introduce a data-driven approach by integrating different types of data. Our modelling approach unveils the relationship between distress and mental disorders propagation and suicidal ideation spreading, shedding light on the role of awareness in a social network for suicide prevention. The proposed model is able to quantify the impact of overlapping awareness on suicidal ideation spreading and our findings demonstrate that it plays a dual role on contagion, either reinforcing or delaying the contagion outbreak.

## Introduction

In recent decades, a large body of literature has attempted to better understand the social contagion, suggesting that a phenomenon spreading in a social network depends on the nature of social ties^1,2^. Behaviours, indirect reciprocity, misinformation or rumors^3^, infectious diseases and emotions^4^ have been found to spread interpersonally^1,2,5^. For this reason, social contagion, modeled as an infectious disease spreading, has been emerging as a growing research field^2,4–7^. These models have been obtained starting from the classical epidemiological models^8–10^, involving several research fields in network science^11–18^. Furthermore, various processes have been used to model social contagion as diffusion models^19^ and threshold models^2,5–7,20^. The interplay between infectious diseases and awareness dynamics has allowed to underline the role of awareness in the spreading process of a disease^21–24^. The more the networked individuals are aware of the likely disease spreading, the more they may be able to adopt strategies targeted at self-protecting^25^. Most of these studies have explored the spreading and competition of both phenomena using different layers of propagation^22,26–30^. Multiplex networks, that consider the same set of nodes in all the layers, constitute the most suitable network structure for studying such dynamical processes and their complex coevolution^31,32^. Although having considered multiplexity, all the previous models have separated and constrained each of the processes to only one of the layers. By contrast, in^25^ it has been investigated and quantified the impact of the coevolution of the two processes in all the layers of a multiplex network. Coherently with the real nature of multiplex networks^33–35^, it has been taken into account heterogeneity and its impact along with awareness on the epidemic spreading^25,36^. Aiming at capturing the complexity of the coevolution, we consider a weighted multiplex network, as social ties between nodes may have different weights reflecting their intensity^37^. We provide a new definition of weight, strongly linked with coevolution of social contagion and awareness spreading, which includes the difference of awareness and homophily between nodes. Starting from^25^, our work is targeted at proposing a model of social contagion coevolving with awareness spreading, by introducing heterogeneity, both in terms of susceptibility and awareness, on each node and layer of a weighted multiplex network.

We define and introduce the novel concept of “overlapping awareness”, that is the co-occurrence of at least two types of awareness, one focused on the primary contagion phenomenon, and the other(s) centered on issues correlated to a certain extent with the main one. Overlapping awareness is a sort of comorbidity, as defined in^38^. We analyse this coevolution dynamics in terms of social contagion and overlapping awareness spreading on weighted multiplex networks, exploring how the phase transitions and contagion thresholds change according to the network structure, which in turns depends on the nodes’ heterogeneity, homophily and overlapping awareness. To validate our analytical model, we adopt a data-driven approach, including real data extracted from multiple sources. We compare our analytical model with simulation results from data referred to suicide contagion^39–41^, in order to demonstrate the coherence with our model. Since social ties and the environment are closely related to people’s health^1^, we investigate the role of social networks in detecting, protecting and understanding influences on mental health, which has been subject to many debates. The connectedness of social networks has been shown to have an impact on depression^42–44^, happiness and mood^6,45^, loneliness^46^, and can represent a tuning parameter of different health outcomes, ranging from obesity to alcohol consumption^1,47,48^. Depression is defined as a common mental disorder, linked with several symptoms^49^. It represents a global health concern, and suicide associated with depression (as estimated by WHO) is the second leading cause of death among young people. This mental ill-health problem continues to be under-diagnosed and many cases still remain undetected^50^, since a person in mental distress could exhibit some symptoms such as anxiety and depression, without being ill. Depression and anxiety have bidirectional interactions with social environment, present a number of biological, psychological and social interacting components, and influence the onset of the illness and its evolution^51^. These disorders have a high level of comorbidity and impact the quality of the social relationships^42,51,52^. Many studies and analysis suggest how depression may shape the vulnerable individual’s social network as well as it may be shaped by its connectedness^42,53^. Social interactions can affect wellbeing and mental health in many ways, impacting on depression symptoms, even though the connectedness of people can lead to less loneliness, greater self-esteem, life satisfaction, greater feeling of belonging. The idea that social and behavioural influences impact on mood symptoms, in particular in the context of suicide, is an old hypothesis^54^. The sociologist Emile Durkheim has highlighted that, although depression and suicide were seen individualistic illness conditions, they could be driven and influenced by social environment and social relationships^54^. In^42^ the authors have explored the possibility of person-to-person spreading of depressive symptoms, demonstrating that they can travel into social networks. To the extent that suicide is a product of voluntary behaviours of vulnerable people, the connectivity among them, according to the type and content of interactions, may increase the exposure to suicide and the risk of being infected by suicidal ideation as a part of suicidal behaviour (suicide ideation, plans and attempt)^55,56^ and a consequence of depression^39,57,58^. In our work we consider the propagation of distress and mental disorders in social multiplex networks and we focus on the suicidal ideation spreading as case study. Since an early detection of suicidal risk factors acts on how we can limit the diffusion of some dangerous conditions, it becomes crucial in terms of prevention^59^. Most of the research on suicide contagion presages the rise of social networks and media^40^ and, consequently, it is crucial to understand how suicide is likely to become more contagious. The spreading process can be influenced by the difference of awareness, the heterogeneity, in terms of susceptibility to the social contagion phenomena, and the homophily. To quantify this process, in this work we consider a weighted multiplex network of vulnerable people, experiencing distress and mental disorders, in which the suicidal ideation can spread as a social contagion phenomenon in conjunction with the spreading of overlapping awareness. To this aim, we introduce weights based on awareness and homophily, as statistical estimators allowing us to quantify their impact on the spreading process. In our data-driven analysis, we deal with estimators and markers about different facets of suicidal ideation spreading, ranging from machine classification datasets for suicide-related communications^41^, Google Trends of suicide keywords and suicide rates from different world countries in two temporal windows, related to the period around a specific suicide event, and suicide rates in the subsequent year. Our interest towards suicidal ideation spreading as social contagion phenomenon is to unveil to what extent the coevolution of contagion and overlapping awareness in a weighted multiplex network can impact on the spreading dynamics, delaying the possible outbreak. On one hand, this allows reducing the vulnerability to suicidal ideation while, on the other hand, it provides a temporal window where interventions in terms of support, information, helpline and prevention can be scheduled. Moreover, we discuss the findings and results of analytical model and data-driven analysis, and future applications in the context of Information and Communication Technology (ICT) for society that highlight the need of the digitalisation of networked people to produce human-related structured data for the future Internet-of-People (IoP)^60^.

## Model

**Figure 1:**
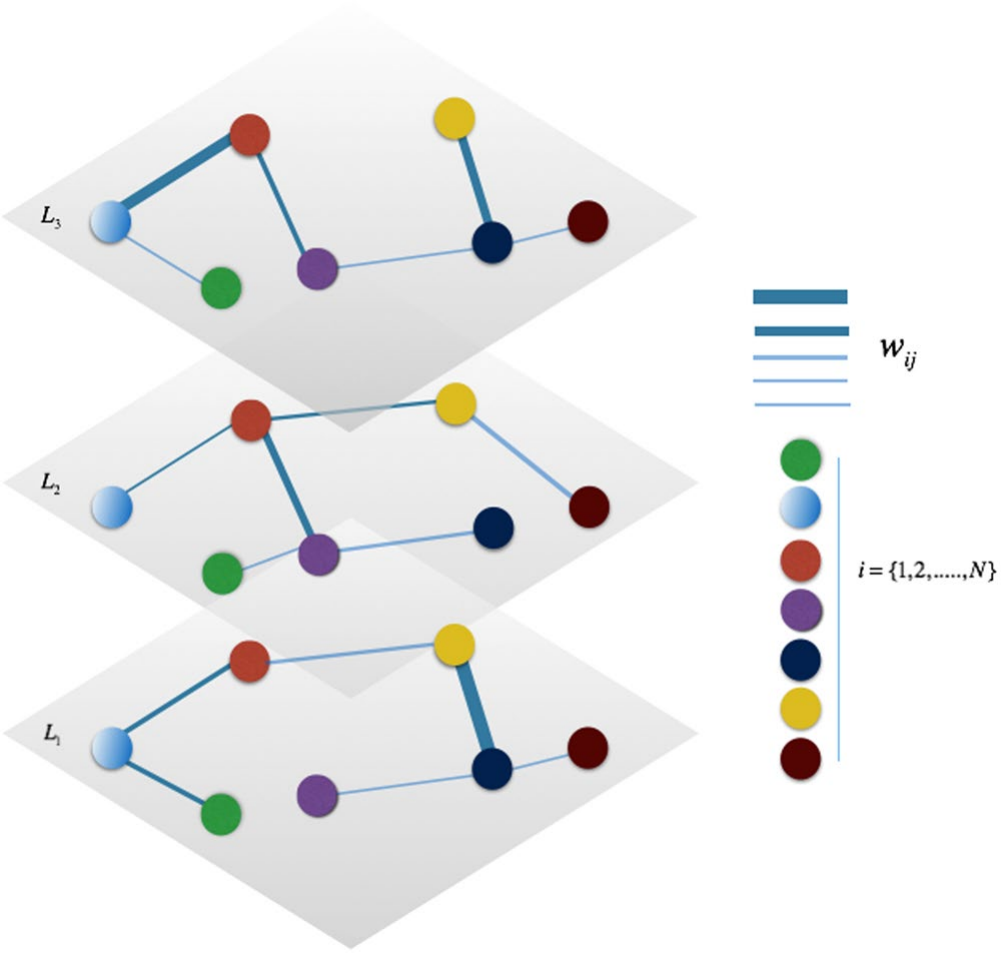
Schematic example of a Weighted Multiplex Network. Colors identify the set of nodes *i* = 1, .., *N*, which is the same for each layer, while the edges’ thick identify the different weights *w*_*ij*_.

In this work, we start from the model presented in^25^, generalizing and extending it. First, as well as in^25^, we consider a SIR-like model, *S*^*h*^*IR*, thought as a “composed” SIR, namely an extension of the classic “Susceptible-Infected-Recovered” (SIR) model^8,13,61^, where *S*^*h*^ represents the heterogeneous susceptibility of each node in the layers of multiplex structure (see eq. 1). The second spreading process, coexisting and coevolving with the first one, is an extension of the “Unaware-Aware-Faded” (UAF)^25^, denoted by *UAF*(*A*^*π*^), where *A*^*π*^ is the “overlapping awareness”, which derives from a non-zero probability *ε* of having an additional awareness correlated to the primary contagion phenomenon (see eq. 2). This represents an alternative state to *F*, as shown in the Dynamic Microscopic Markov Chain Approach (MMCA) (see details in Methods). This means that a node which is in the awareness state *A* may decide to acquire an awareness on another issue related to the primary contagion process, thus adding an extra awareness, rather than having a transition to the fading state *F*, where instead a node have a tendency to fade its attention over time until it completely vanishes. Differently from^25^, for the first time we consider a dual heterogeneity of nodes’ susceptibility and awareness in the layers of the weighted multiplex network. This results in a variation of the infection rate ${\beta }_{i}^{\alpha }$ and the rate of awareness ${\lambda }_{i}^{\alpha }$ for the generic node *i* at layer *α*, with *α* ∈{1, ..., *M*}. In this work, we decide to consider weighted multiplex networks as network structure, and the heterogeneous factors, included in the analytic definition of the infection rate and the rate of awareness, are obtained from properties of the weighted multiplex networks^37^ (see details in Model). Heterogeneity and overlapping awareness are introduced in this model in order to describe a realistic spreading scenario and disentangle the complex coevolution of two interdependent processes, the social contagion and the awareness spreading on the contagious phenomenon, without neglecting the crucial influence of other aspects related to the contagion. Let us consider a weighted multiplex network of *M* layers *α* = {1, ..., *M*} and *N* nodes *i* = {1, ..., *N*}, which is a set of *M* weighted networks *G*_*α*_ = (*V*, *E*_*α*_) (see Fig. 1). The set of nodes *V* is the same for each layer, whereas the set of links *E* changes according to the layer^37,62^. Each network *G*_*α*_ is described by the adjacency matrix, denoted by *a*^*α*^ with elements ${a}_{ij}^{\alpha }$, where ${a}_{ij}^{\alpha }={w}_{ij}^{\alpha } > 0$, if there is a link between *i* and *j*, with a weight *w*_*ij*_, otherwise ${a}_{ij}^{\alpha }=0$. The heterogeneity of weights’ distribution in the multiplex network can be evaluated by means of the two following local properties^37,62^: the strength of nodes, ${s}_{i}^{\alpha }$, that is the sum of the weights of the links incident upon node *i* in layer *α*, and the inverse participation ratio, ${Y}_{i}^{\alpha }$, which indicates how the weights are distributed in the layer *α*^37,62^.

**Figure 2:**
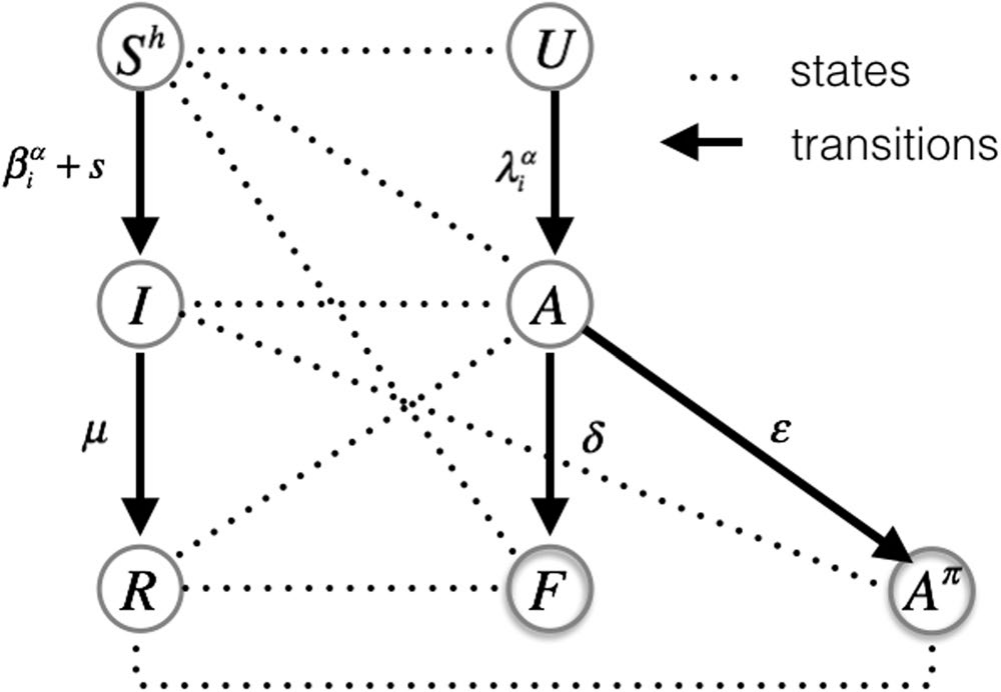
Coevolution of social contagion and awareness spreading *S*^*h*^*IR* − *UAF*(*A*^*π*^) - States and transitions. *S*^*h*^ heterogeneous susceptibility state; *I* infected state; *R* recovered state; *U* unaware state; *A* aware state; *F* faded state; *A*^*π*^ overlapping aware state; ${\beta }_{i}^{\alpha }$ infection rate; *μ* recovery rate; ${\lambda }_{i}^{\alpha }$ rate of awareness; *δ* fading rate; *ε* rate of overlapping awareness; *s* spontaneous contagion.

In our model, we consider the coevolution of two spreading processes on a weighted multiplex network (see Fig. 2). The first is the process of social contagion spreading, *S*^*h*^*IR*, which is a *SIR*-like model^8,9^, where *S*^*h*^ indicates heterogeneous susceptible state^25^, which means that each node has a different infection rate *β*_*i*_ (see eq. 5). As second spreading process, we consider the *UAF*(*A*^*π*^) model, SIR-like, that is the “Unaware - Aware – Faded/Overlapping Aware”, which is an extension of the *UAF* model^25^, where *U* indicates the condition of unawareness, *A* is the aware state where nodes begin to have an interest in the social contagion phenomenon, increasing their attention, while in the *F* state, nodes tend to decrease their attention over time up to the point that it completely vanishes. When a node reaches this state, it maintains the same awareness, but it has no interest in increasing its acquired awareness on the phenomenon. The more susceptible are nodes that reach the faded state, the more vulnerable they become due to their low resilience against the phenomenon. Alternatively, if they have a transition from *A* to *A*^*π*^, an alternative state to *F*, they have the opportunity to increase their awareness also about other issues correlated with the primary contagion phenomenon.

1 $$\begin{array}{l}{S}^{h}IR\Rightarrow {S}^{h}\mathop{\to }\limits^{{\beta }_{i}^{\alpha }}I\mathop{\to }\limits^{\mu }R;\end{array}$$

2 $$UAF({A}^{\pi })=\{\begin{array}{cc}U\mathop{\to }\limits^{{\lambda }_{i}^{\alpha }}A\mathop{\to }\limits^{\delta }F, & if\quad \varepsilon =0\\ U\mathop{\to }\limits^{{\lambda }_{i}^{\alpha }}A\mathop{\to }\limits^{\varepsilon }{A}^{\pi },\, & otherwise\end{array}$$

We introduce a new definition of weight in the multiplex network, as follows: ${w}_{ij}^{\alpha }={h}_{ij}^{\alpha }\cdot |a{w}_{i}-a{w}_{j}|+1$. Weights are function of *h*_*ij*_, which is the homophily between nodes, that is the tendency to associate and interact more with similar people^34,35^, and the absolute difference of awareness, |*aw*_*i*_ − *aw*_*j*_|, between nodes *i* and *j*. Thus, when this difference of awareness is equal to zero, nodes will have a weight ${w}_{ij}^{\alpha }=1$, only if there is a link between *i* and *j*. Homophily is defined as follows:

3 $$\begin{array}{l}{h}_{ij}^{\alpha }=\frac{1}{1+{\delta }_{ij}^{\alpha }}\end{array}$$

where ${\delta }_{ij}^{\alpha }$ is the measure of the homophily difference between nodes *i* and *j*. To bind this type of weighted network structure with the coevolving spreading processes, showed in eq. 1 and 2, we define the rate of awareness, ${\lambda }_{i}^{\alpha }$, and the infection rate, ${\beta }_{i}^{\alpha }$, for each node *i* at each layer *α* of the multiplex, as follows:

4 $$\begin{array}{l}\begin{array}{l}{\lambda }_{i}^{\alpha }={\gamma }_{i}^{\alpha }\lambda \end{array}\end{array}$$

5 $$\begin{array}{l}\begin{array}{l}{\beta }_{i}^{\alpha }={{\psi }}_{i}^{\alpha }\beta +s\end{array}\end{array}$$

The rate of awareness and the infection rate are interdependent since ${\beta }_{i}^{\alpha }$ depends on ${\lambda }_{i}^{\alpha }$ (see eq. 7)^25^. Both rates are characterised by the heterogeneous factors, ${\gamma }_{i}^{\alpha }$ and ${\psi }_{i}^{\alpha }$, defined as follows:

6 $$\begin{array}{c}{\gamma }_{i}^{\alpha }=\frac{{s}_{i}^{\alpha }}{1+{s}_{i}^{\alpha }}\end{array}$$

7 $$\begin{array}{c}{\psi }_{i}^{\alpha }=\frac{1}{1+{\lambda }_{i}^{\alpha }}\cdot \frac{1}{{Y}_{i}^{\alpha }}\end{array}$$

In eq. 5 we indicate with *s* the spontaneous contagion, which evaluates the realistic condition to contract the contagion spontaneously regardless the interactions on the whole multiplex network^4^. We define the awareness matrix Λ, where each element is calculated based on eq. 4, as follows:

8 $${\rm{\Lambda }}=[\begin{array}{llll}{\lambda }_{1}^{1} & {\lambda }_{1}^{2} & \mathrm{..}. & {\lambda }_{1}^{M}\\ {\lambda }_{2}^{1} & {\lambda }_{2}^{2} & \mathrm{..}. & {\lambda }_{2}^{M}\\ \mathrm{..}. & \mathrm{..}. & \mathrm{..}. & \mathrm{..}.\\ {\lambda }_{N}^{1} & {\lambda }_{N}^{2} & \mathrm{..}. & {\lambda }_{N}^{M}\end{array}]\,\in {{\mathbb{R}}}^{N\times M}$$

and, the matrix *B*, whose elements are the infection rate for each node in each layer (see eq. 5).

9 $$B=[\begin{array}{llll}{\beta }_{1}^{1} & {\beta }_{1}^{2} & \mathrm{..}. & {\beta }_{1}^{M}\\ {\beta }_{2}^{1} & {\beta }_{2}^{2} & \mathrm{..}. & {\beta }_{2}^{M}\\ \mathrm{..}. & \mathrm{..}. & \mathrm{..}. & \mathrm{..}.\\ {\beta }_{N}^{1} & {\beta }_{N}^{2} & \mathrm{..}. & {\beta }_{N}^{M}\end{array}]\,\in {{\mathbb{R}}}^{N\times M}$$

In the second process spreading process, *UAF*(*A*^*π*^), we introduce an alternative state *A*^*π*^, where if *π* = 1 the awareness is only referred to the primary contagion phenomenon. In the presence of variously correlated issues with the main contagion process, we define the overlapping awareness as follows:

10 $$\begin{array}{l}\begin{array}{l}\overline{a{w}_{i}}=\mathop{\overbrace{{a{w}_{i}|}_{\pi \mathrm{=1}}}}\limits^{awareness}+\mathop{\overbrace{\sum _{\pi \mathrm{=2}}^{T}a{w}_{i}^{\pi }}}\limits^{awareness\,on\,correlated\,\,issues}\end{array}\end{array}$$

with $a{w}_{i}^{\pi }={\varphi }_{1,\pi }\cdot a{w}_{i}$, where *ϕ*_1,*π*_ is the *ϕ*-correlation between the primary contagion phenomenon and the other issues on a space of issues *T*. Based on the previous definition of overlapping awareness, the ${w}_{ij}^{\alpha }$ becomes:

11 $$\begin{array}{l}{w}_{ij}^{\alpha }={h}_{ij}^{\alpha }\cdot \mathop{\overbrace{|\overline{a{w}_{i}}-\overline{a{w}_{j}}|}}\limits^{difference\,\,of\,awareness}+1\end{array}$$

considering also the awareness on *T*. In order to capture the potential heterogeneity of the network structure in terms of weights, we introduce a measure of centrality of both nodes and layers, *X*_*i*_ and *z*^*α*^ as defined in^63^, to obtain the simultaneous ranking of nodes and layers.

These measures are coupled to get a simultaneous ranking of nodes *X*_*i*_ and layers *z*^*α*^, an overall measure of centrality for nodes and layers. In our model, it is dependent on the weights of the multiplex network, therefore including awareness and homophily (see Supplementary Figure S1). We exploit this kind of measures because we apply a rewiring process^64^, in which we choose the fraction of the links to be rewired considering the less central nodes in the less central layer, based on the previously defined ranking (see Simulation Results).

## Results

### Simulation Results

**Figure 3:**
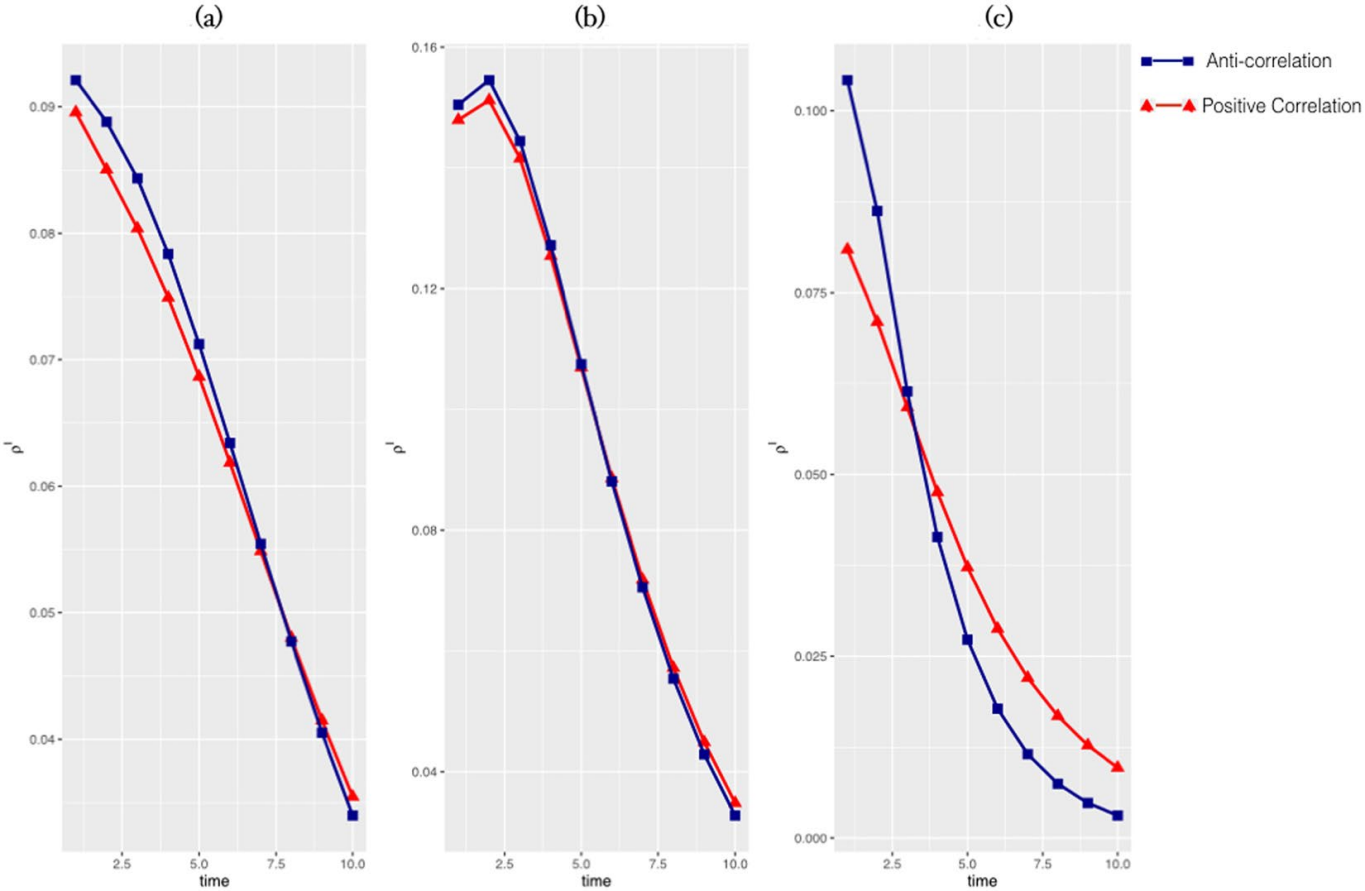
Density of infected nodes over time. We show the temporal evolution of the density of infected nodes *ρ*_*i*_ in function of *ϕ* (see eq. 10), with the following parameters of the initial rates: (**a**) *μ*,*δ* = 0.3; *ε* = 0.7; *ϕ* = −0.8 (red); *ϕ* = 0.8 (blue); (**b**) *μ* = 0.3; *δ* = 0.7; *ε* = 0.3; *ϕ* = −0.8 (red); *ϕ* = 0.8 (blue); (**c**) *μ*,*δ* = 0.7; *ε* = 0.3; *ϕ* = −0.8 (red); *ϕ* = 0.8 (blue).

**Figure 4:**
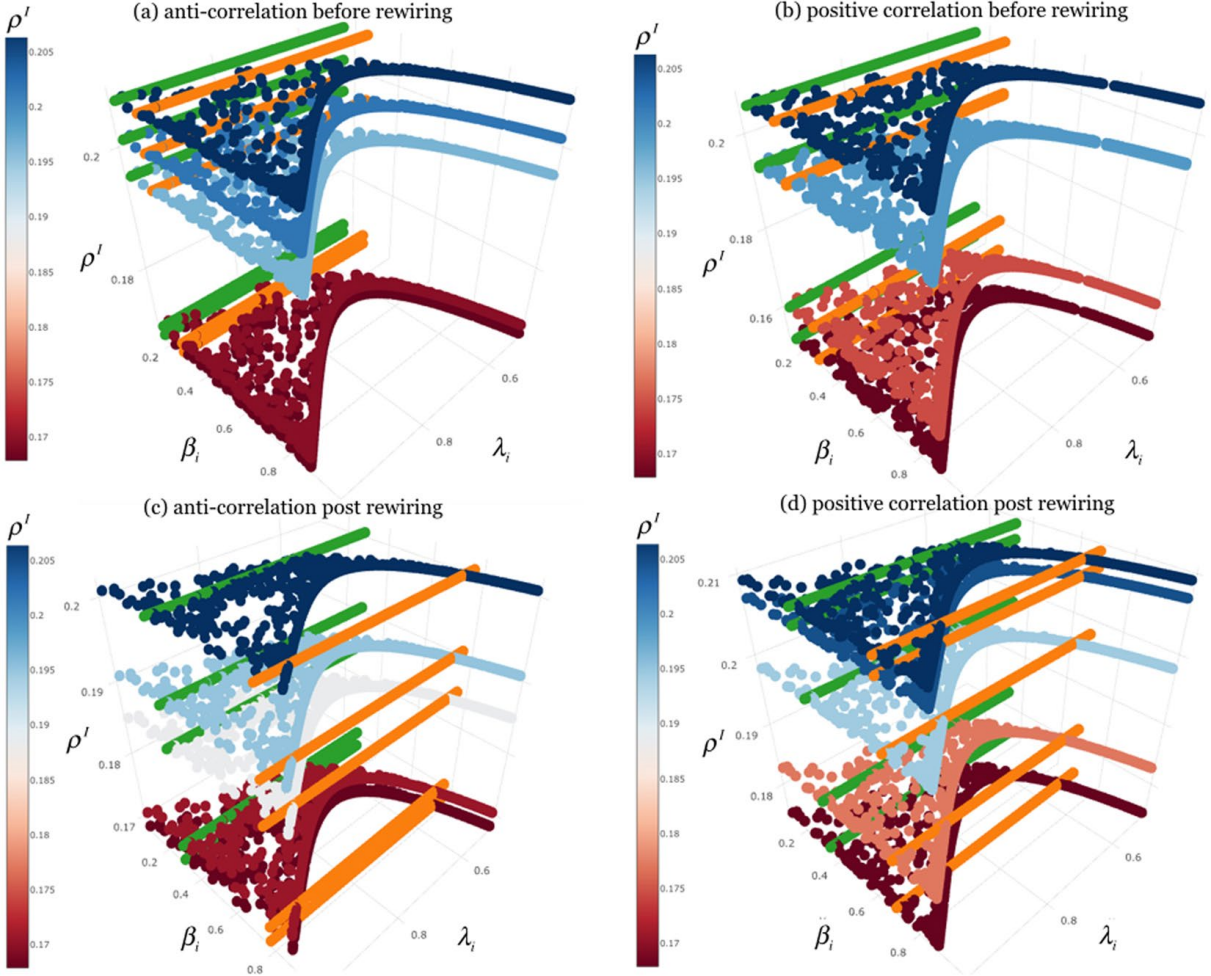
Phase diagrams of social contagion and overlapping awareness on weighted multiplex network. The plots show the density of infected nodes *ρ*^*I*^ (see color on z-axis, from red to blue, as in color bar in the middle) according to the rate of awareness *λ*_*i*_ (x-axis) and the infection rate *β*_*i*_ (y-axis). We compare the contagion threshold obtained in the homogeneous susceptibility case (‘green’) and that one derived from our model (‘orange’). In (**a**) there is the phase diagram with *ϕ* = −0.8, (anti-correlation case, before rewiring). In (**b**) we show the phase diagram with a positive correlation *ϕ* = 0.8 (positive correlation case, before rewiring). In (**c**) we show the phase diagrams after the rewiring of the links of the nodes, in the anti-correlated case (anti-correlation case, post rewiring) and in (**d**) the positive correlation case (positive correlation case, post rewiring).

**Figure 5:**
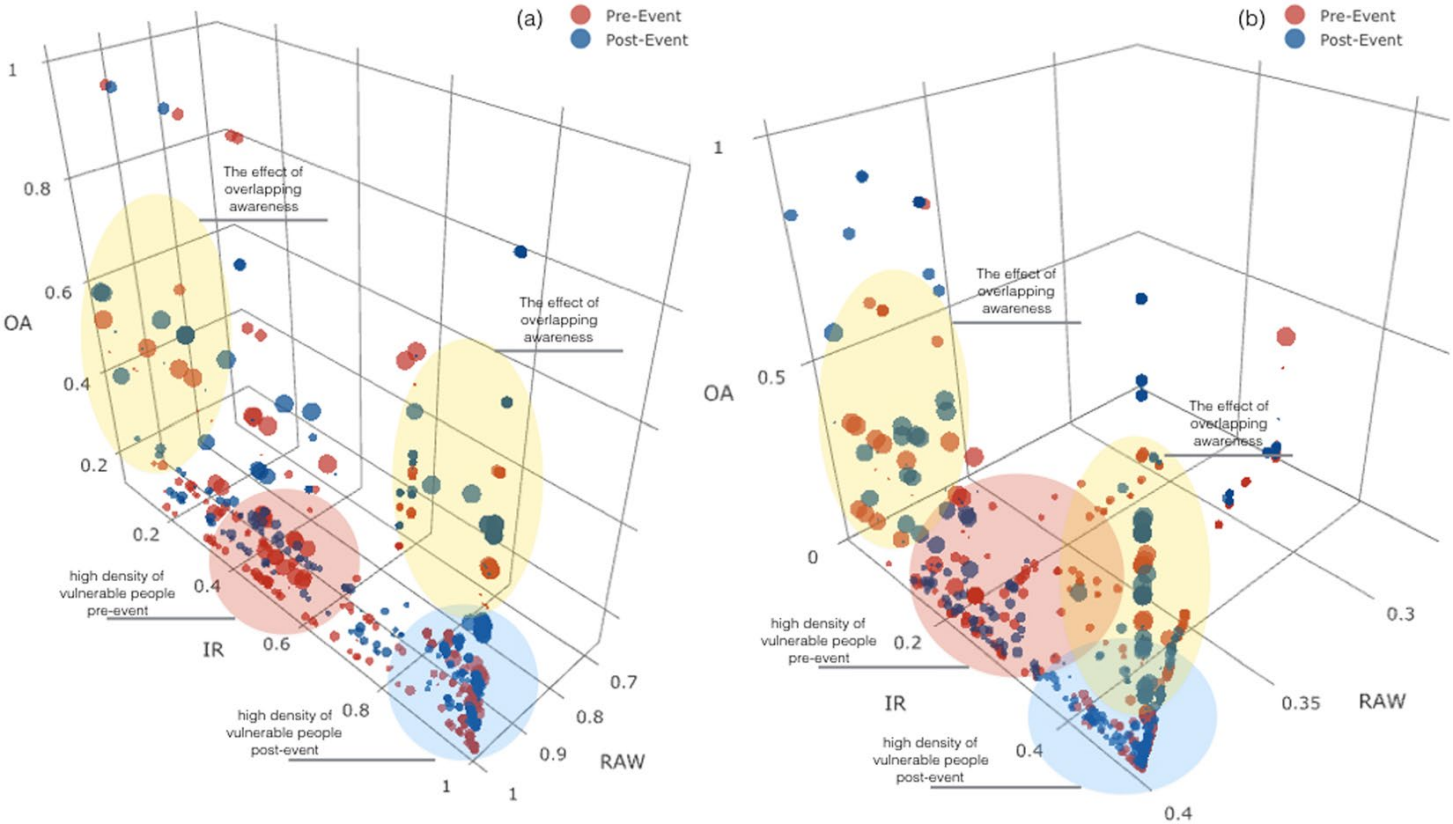
Data-driven analysis in the plane *λ*_*i*_ (*RAW* - Rate of Awareness), *β*_*i*_ (*IR* - Infection Rate), $\overline{a{w}_{i}}$ (*OA* - Overlapping Awareness) for the two keywords ‘suicide’ (**a**) and ‘suicide prevention’ (**b**). We illustrate how the rate of awareness *λ*_*i*_ (x-axis) and the infection rate *β*_*i*_ (y-axis) change according to the measure of overlapping awareness derived from data $\overline{a{w}_{i}}$ (z-axis). The size is the awareness measure according to the associated class (see details in Methods), where small nodes are the most vulnerable. In both plots, data are derived from the searches on terms ‘suicide’ (**a**) and ‘suicide prevention’ (**b**) (for the sake of clarity the plot has been zoomed-in in order to visualize areas covered by dots). We show the temporal evolution of the rates in the time window referred to two months before an event suicide (‘red’ dots) and that one referred to the two months subsequent to an event suicide (‘blue’ dots). Red circle and blue circle highlight the area with a high density of more vulnerable people, in the pre-event case and in the post-event case, respectively. Yellow circles highlight the effect of overlapping awareness.

Simulations have been carried out considering a multiplex network with *M* = 3 layers, where each layer is modeled as a scale-free network^65^ with *N* = 1000 nodes. In Fig. 3, each curve corresponds to a different value of the *ϕ*-correlation of the primary contagion phenomenon with the other issue, in both cases of anti-correlation and positive correlation. The plots show how the density of infected nodes depends on to what extent the specific issue is correlated with the social contagion of the primary phenomenon. In (a), where nodes maintain a high attention to the contagion (see details in Model), we can observe how the density of infected nodes for an anti-correlated issue is lower than the case of a positively correlated issue. This extremely interesting result is due to the fact that exceeding in information on issues positively correlated to the contagion phenomenon may produce a negative influence on it, in fact encouraging the contagion rather than curbing it. In (b) nodes’ attention to contagion fades quickly over time, so this vanishes the effect of correlation and the density of infected nodes in the two cases of anti-correlation and positive correlation results approximately the same. Finally, in (c), the two dynamics are close and the high probability of getting into the faded state causes a scarce interest in the main contagion. It produces an overall decrease in the density of infected and in some points the anti-correlated curve is better than the positive correlated one because the dynamics after contagion is faster. In Fig. 4, we show how the double heterogeneity, in terms of both infection rates and rates of awareness, allows delaying the contagion outbreak compared to the homogeneous case, where nodes have a uniform susceptibility and rate of awareness. Comparing the phase diagrams before and after applying the rewiring, we can observe that the contagion threshold is more delayed in the post-rewiring cases, as we expected. Overall, the gap among the contagion thresholds between homogeneous and heterogeneous cases is wider in the anti-correlated case. In other words, the figure highlights the effect due to the presence of overlapping awareness, depending on the type of correlation with the primary contagion phenomenon. Although the impact is overall positive delaying the threshold, it is more evident in the anti-correlated case. In Fig. 5, we show the results of the data-driven approach with regards to a population of nodes (see details in Methods), according to the data on suicidal ideation spreading, taking into account the two temporal windows before (pre-event) and after a specific suicide event (post-event). In (a), where the overlapping awareness is referred to a positive correlated keyword, the more vulnerable nodes (small-sized nodes) show a high infection rate, and this is more evident in the post-event case, as highlighted by blue circle, as the rate of awareness increases. The red circle emphasises the area with more vulnerable people in the pre-event case, while the yellow circles show the effect of the overlapping awareness’ increasing. In (b), in the case of anti-correlated keyword, the overall infection rate is lower than the previous case, and as the rate of awareness increases, the distribution of the more vulnerable nodes remains confined in a region of low infection rates. This means that, differently from the previous case, the rate of awareness does not boost the contagion, but bounds the more vulnerable people within a range of low infection rates, thanks to the spread of positive contents, such as prevention, related to suicide. By using the red circle and the blue circle we highlight the high density area of vulnerable people in the pre-event case and post-event case, respectively. Yellow circles underline the effect of the overlapping awareness. We shed light on how the overlapping awareness apparently could act on the less vulnerable people (high-sized nodes), but influences the overall network, through social contagion dynamics. This demonstrates the dual role of overlapping awareness in the case of a social contagion phenomenon, such as suicidal ideation (see Discussion).

## Methods

### Dynamic Microscopic Markov Chain

**Figure 6:**
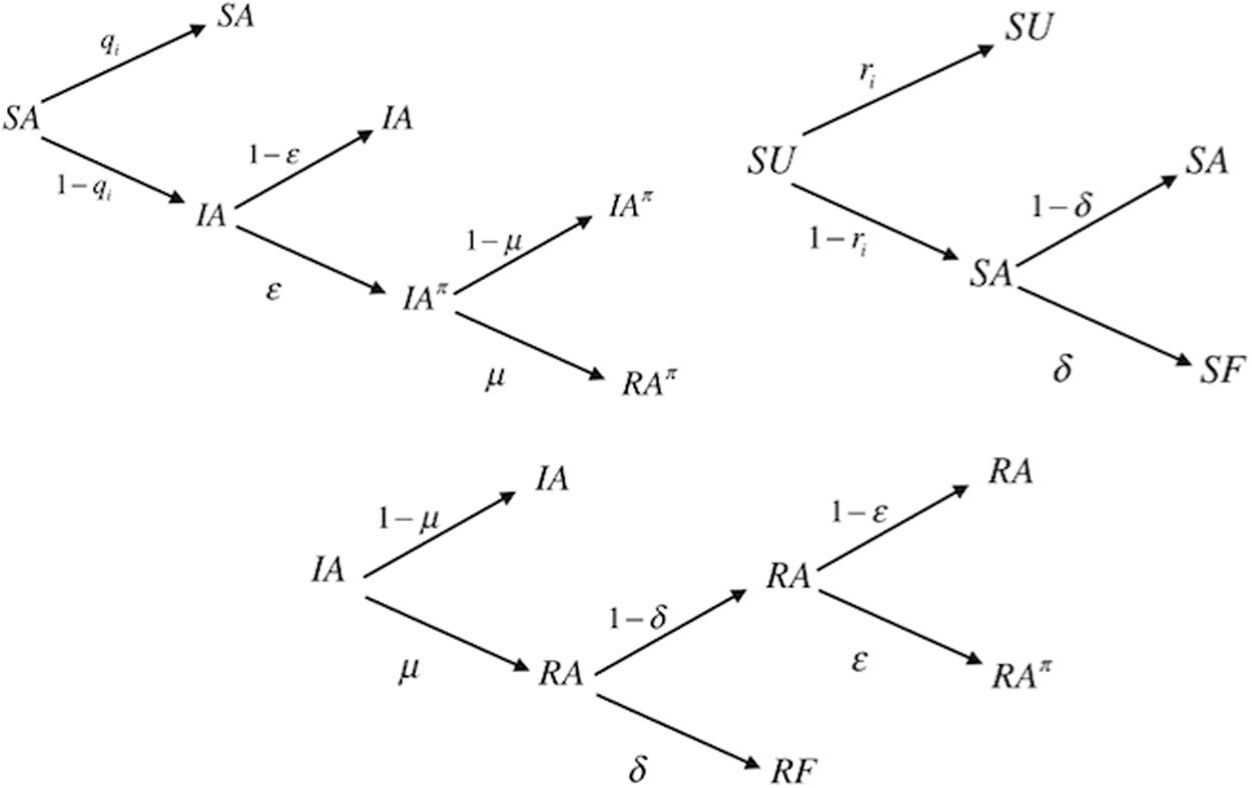
Probability tree. We illustrate the MMCA method using a probability tree, representing all the possible states and their transitions in our model at each time step. Roots in the transition tree represent the initial states (time step *t*), *SA*, *SU* and *IA*, leaves are all the possible states at the subsequent time step. Arrows are labeled with the corresponding transition probabilities.

To explore the dynamics of the coevolution of social contagion and awareness spreading on the weighted multiplex network, we take into account the Dynamic Microscopic Markov Chain Approach (MMCA). Initially, we assign to each node a state probability to be in one of the initial states. At the beginning, each node in the weighted multiplex network can occupy only one of the following states: susceptible and unaware (SU), infected and aware (AI), and susceptible and aware (SA). Some states are not reachable or do not exist, such as *IU* (Infected Unaware), *IF* (Infected Faded), *SA*^*π*^ (Susceptible - Overlapping Aware) and *FA*^*π*^ (Faded - Overlapping aware) (see Fig. 6). At time step *t* each node *i* can occupy one of the initial three states, with probabilities ${p}_{i}^{SU}(t)$, ${p}_{i}^{SA}(t)$ and ${p}_{i}^{IA}(t)$ respectively. Moreover, we define: *q*_*i*_(*t*), probability of node *i* not being infected at time step *t* and *r*_*i*_(*t*), probability of unaware node *i* staying unaware at time step *t*, as follows:

12 $$\begin{array}{l}{q}_{i}(t)=\mathrm{(1}-{\bar{\beta }}_{i})\prod _{j}\mathrm{[1}-{a}_{ji}{p}_{j}^{I}(t){\bar{\beta }}_{i}]\end{array}$$

13 $$\begin{array}{l}{r}_{i}(t)=\mathrm{(1}-{\bar{\lambda }}_{i})\prod _{j}\mathrm{[1}-{a}_{ji}{p}_{j}^{A}(t)\overline{{\lambda }_{j}]}\end{array}$$

where *a*_*ij*_ are the elements of the adjacency matrix of each layer of the weighted multiplex network. ${\bar{\beta }}_{i}$ and ${\bar{\lambda }}_{i}$ are the “elected infection rate” and the “elected rate of awareness” of the node *i*, respectively. Once calculated the centrality measures of nodes and layers *X*_*i*_ and *z*^*α*^, from this heterogeneous ranking we extract the “elected” layer, that is the most central layer and in both matrices *B* and Λ, we select the corresponding column. We consider the most central layer because it is the most influential in the evaluation of the transition dynamics. The following MMCA equations represent the probability of each node of being in one of the states at time step *t* + 1, as showed in Fig. 6:

14 $$\begin{array}{rcl}{p}_{i}^{SA}(t+1) & = & {q}_{i}(t){p}_{i}^{SA}(t)+(1-{r}_{i}(t))(1-\delta ){p}_{i}^{SU}(t);\\ {p}_{i}^{IA}(t+1) & = & (1-{q}_{i}(t))(1-\varepsilon ){p}_{i}^{SA}(t)+(1-\mu ){p}_{i}^{IA}(t);\\ {p}_{i}^{I{A}^{\pi }}(t+1) & = & \varepsilon (1-{q}_{i}(t))(1-\mu ){p}_{i}^{SA}(t);\\ {p}_{i}^{R{A}^{\pi }}(t+1) & = & \mu \varepsilon (1-{q}_{i}(t)){p}_{i}^{SA}(t)+\mu \varepsilon (1-\delta ){p}_{i}^{IA}(t);\\ {p}_{i}^{SU}(t+1) & = & {r}_{i}(t){p}_{i}^{SU}(t);\\ {p}_{i}^{SF}(t+1) & = & \delta (1-{r}_{i}(t)){p}_{i}^{SU}(t);\\ {p}_{i}^{RA}(t+1) & = & \mu (1-\delta )(1-\varepsilon ){p}_{i}^{IA}(t);\\ {p}_{i}^{RF}(t+1) & = & \mu \delta {p}_{i}^{IA}(t);\end{array}$$

To obtain the contagion threshold, we explore the steady state solution of the system constituted by the previous equations. When time *t* → +∞, there exists a contagion threshold *β*_*C*_ for the two coevolving processes, so that the contagion can outbreak only if *β* ≥ *β*_*C*_. Following the same conditions of^25^, the contagion threshold is given by the order parameter *ρ*_*i*_ and it is defined as follows:

15 $$\begin{array}{l}{\rho }^{I}=\frac{1}{N}\sum _{i\mathrm{=1}}^{N}{p}_{i}^{I}=\frac{1}{N}\sum _{i\mathrm{=1}}^{N}{p}_{i}^{IA}\end{array}$$

Thus, starting from equation ${p}_{i}^{IA}(t+\mathrm{1)}$ (see eq. (14)), at steady state we have:

16 $$\begin{array}{l}{p}_{i}^{IA}=(1-{q}_{i})(1-\varepsilon ){p}_{i}^{SA}\end{array}$$

Since around the contagion threshold *β*_*C*_, the infected probability is close to zero (${p}_{i}^{IA}={\eta }_{i}\ll 1$), the probabilities of being infected can be approximated as follows:

17 $$\begin{array}{l}{q}_{i}=(1-\overline{{\beta }_{i}})[1-\overline{{\beta }_{j}}\sum _{j}{a}_{ij}{\eta }_{j}]=(1-\overline{{\beta }_{i}})(1-{\omega }_{i})\end{array}$$

where:

18 $$\begin{array}{l}{\omega }_{i}=\overline{{\beta }_{j}}\sum _{j}{a}_{ij}{\eta }_{j}\end{array}$$

Furthermore, close to the contagion onset we have that the fading rate is approximately close to zero ($\delta \simeq 0$). Considering this approximation into eq. 16 and omitting higher order items, equation 16 is reduced to the following form:

19 $$\begin{array}{l}\begin{array}{l}\mu {\eta }_{i}\simeq (1-\varepsilon ){p}_{i}^{SA}\overline{{\beta }_{i}}\overline{{\beta }_{j}}\sum _{j}{a}_{ij}{\eta }_{j}\end{array}\end{array}$$

The contagion threshold is obtained starting from the following condition:

20 $$\begin{array}{l}\sum _{j}|(1-\varepsilon )\overline{{\beta }_{i}}{p}_{i}^{SA}{a}_{ij}-\frac{\mu }{\overline{{\beta }_{j}}}{t}_{ji}|{\eta }_{j}=0\end{array}$$

where *t*_*ji*_ are the elements of the Identity matrix. By defining the matrix *H* whose elements are given by: ${h}_{ij}=[(1-\varepsilon )\overline{{\beta }_{i}}{p}_{i}^{SA}]{a}_{ij}$, the contagion threshold *β*_*c*_ is the one that satisfies that Λ_max_(*H*), the largest eigenvalue of the matrix *H* is given by ${{\rm{\Lambda }}}_{\max }(H)=\mu /\overline{{\beta }_{j}}$, and finally we get: *β*_*c*_ = *μ*/Λ_max_(*H*).

### Data-driven analysis

In our model, we consider a data-driven approach for evaluating the overlapping awareness, which is the result of the different types of awareness on suicidal ideation spreading as a social contagion phenomenon^66,67^. First, we consider data derived from a machine classification dataset for suicide-related communications, where classes represent the types of suicidal communication with relative percentage proportion in dataset^41,68^. We decide to construct our population of *N* = 400 nodes based on these classes^41,68^ which represents the best representation of how people generally communicate on the topic of suicide. We associate an awareness score to each node which depends on three measures. The first measure is related to a distinct probability to post a text according to the associated class, that is an initial measure of awareness ranging from a low level to a high level. The second measure is associated with the Google search popularity of terms related to the classes of two geographical countries (see Supplementary Table S1, Figures S2, S3). Homophily corresponds to the geographical proximity of nodes, so that two individuals of the same country will have a high homophily. The third measure relates to the searches on Google Trends on issues either positively or anti-correlated with the primary contagion. Google Trends allows evaluating the time evolution of awareness and setting up a measure related to the interest in specific aspects of suicide contagion. In particular, we keep track of the total Google Trends search-volume of some of the most significant suicide keywords, such as ‘suicide’ and ‘suicide prevention’, in two temporal windows related to the period around a specific suicide event. We aim at shedding light on how these searches pre-event suicide and post-event suicide contribute to the contagion dynamics. The temporal window is that one around the Robin Williams’ suicide, occurred on August 11, 2014, so the two temporal windows before and after the event suicide are respectively from June 10, 2014 to August 10, 2014, and from August 12, 2014 to October 10, 2014. The target is to analyse the temporal evolution of the overlapping awareness, consisting of an aggregated measure of these sources. Furthermore, in order to extend our understanding on the importance of the Google Trends on the awareness about the suicide contagion, we choose three keywords, comparing the Google search popularity in different countries across the world of these terms in the subsequent year of the suicide event with the suicide rates of the same countries (see Supplementary Figure S3).

## Discussion

**Figure 7:**
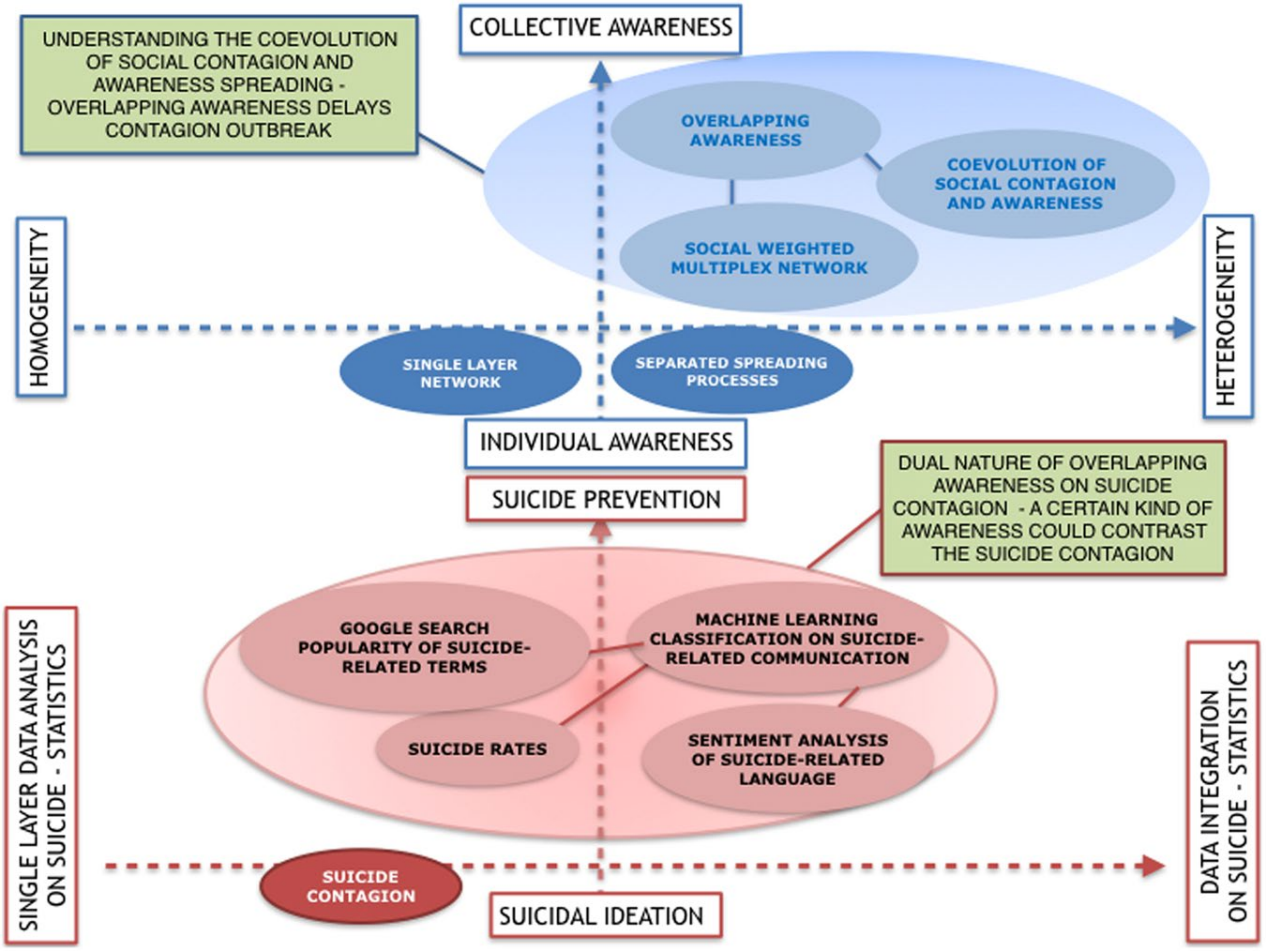
Awareness and Suicide Contagion. The figure depicts the scenarios we deal with in this work. The axis in blue highlights how we can pave the way to obtain collective awareness and heterogeneity. The axis in red show how to obtain suicide prevention strategies and data integration on suicide. The figure summarises each aspect we focused on in our model and data-driven analysis, in order to understand the coevolution of overlapping awareness and suicide contagion. Awareness, heterogeneity and multiplexity are the key factors to shed light on how to face with a contagion phenomenon. In green boxes, we highlighted the main findings.

**Figure 8:**
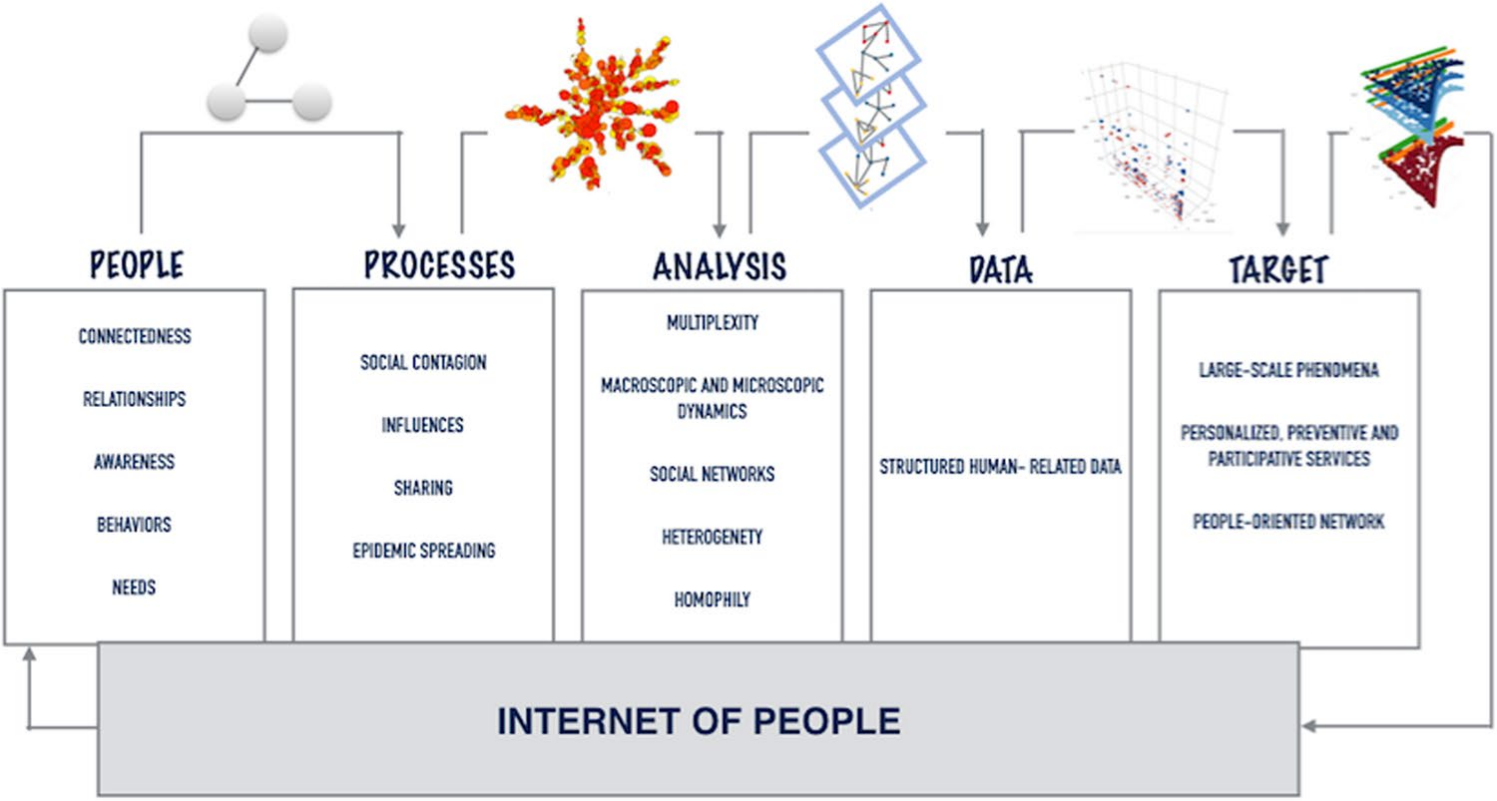
Internet-of-People Framework: a smart and digital corpus of solutions that takes into account connectedness, sharing and social environment to obtain a people-oriented network giving personalised, participative and preventive services through human-related structured data.

Connectedness among people is deeply involved in the spreading phenomena in real-world networks. Influences, awareness, ideas travel through the same multiple interaction channel, impacting each other. To capture and quantify the complexity of such dynamics, we propose the coevolution of social contagion and overlapping awareness spreading in weighted multiplex networks. We quantify the propagation of distress and mental disorders that may lead to suicidal ideation spreading, which is one of the most challenging and less understood aspects of suicide^69–71^. To discover whether or not the awareness changes the exposure to suicide, we consider the spreading of suicidal ideation as a case study. Human thinking about the presence of an idea spreading through a realistic social network is bound to subjective awareness, interaction with similar people and the occurrence of a similar awareness among who often share some sort of proximity. In this work, this concept of awareness has been expressed as an overlapping awareness. Our work has proposed a novel model to analyse and quantify the coevolution of social contagion and overlapping awareness spreading on a weighted multiplex network, introducing a double heterogeneity, both in terms of infection rate and rate of awareness, quantified starting from structural measures of the weighted multiplex network. The weights of the interaction between nodes derive from homophily, a measure of their similarity, and the difference of awareness on contagion phenomenon in the multiplex network. We assume not to study the spread of information that gives benefits to society reaching people in a few minutes, but rather how vulnerable people come to harm when a contagion phenomenon spreads a negative ideation, such as misinformation or rumors, suicidal ideation, cyberbullying^1–5,72^. In our model, heterogeneity and weighted multiplexity, increase the resilience of the social network against this kind of phenomena, delaying the contagion outbreak. By applying the rewiring of the connectivity in the weighted multiplex network, this results even more clear, reinforcing heterogeneity in the overall network. In other words, we introduce a realistic perturbation on connectivity which changes the complex dynamics of the coevolution of the two spreading processes. Our findings demonstrate how the overlapping awareness, if anti-correlated with the main phenomenon, plays a key role in delaying the social contagion. Adding a data-driven approach we aimed at exploring how in a real contagion phenomenon influenced by social media and networks^39–41,57,58,73,74^, that of suicidal ideation, the overlapping awareness impacts on its dynamics. In this work, we shed light on the dual nature of the awareness spreading coevolving with the suicide contagion. In fact, an overlapping awareness, such as reporting of suicide, suicide details, amplifying the ideas of suicide, reinforces the contagion effect rather than slowing it. Instead, a different overlapping awareness, such as suicide prevention, social campaigning and helplines, may reduce the suicidal ideation contagion, avoiding possible tragic suicide triggers. The role of awareness may become crucial in heading off vulnerable people before having been triggered by suicidal ideation. Therefore, our model represents a key step forward to better understand the complex dynamics of the coevolution of suicide contagion and awareness spreading in a realistic scenario thanks to the weighted multiplexity. In Fig. 7, we illustrate the scenarios and the key factors, awareness, heterogeneity and multiplexity, included in our work applied to suicide contagion. For the sake of clarity, we have joined the distinct aspects of our model and the main results. Our findings show how a certain kind of awareness could contrast the social contagion of suicidal ideation, improving the suicide prevention strategies. The delay in the contagion outbreak may allow providing real-time support through social networks and media to help deter vulnerable people, who already have suicidal tendencies, from acting on suicidal ideation in response to an excessive increase of information about suicide. The role of social networks is even more important after disaster events (mass shooting, etc.), creating disorder-specific patterns and long-term distress^75,76^. This has been further proved by the results obtained through the data-driven approach. Starting from our results, a future challenge may be the early detection of undiagnosed cases and people unaware of their mental health status. By taking into account that if people are connected also their health is connected in multiple contexts and layers, the target will be to write innovative future policies and design future research based on a new framework (Fig. 8) by using human-related structured data, to deepen understanding of different issues of our society. The technological challenges represent a key factor in achieving reliability and sustainability of the information and communication systems for society. The new target is to put people in the center of these systems for giving the right accessibility to everybody. Today, we can collect, store and analyse big data of a multitude of people, and this allows us to design people-oriented networks. For this reason, Internet-of-People (IoP) refers to the digitalisation of interpersonal relationships and interactions with the aim of storing and analysing personal data^60^. The collective awareness, the social contagion phenomena and spreading processes, other than the sharing mechanisms between digital people, will lead to a novel and interesting target to support and design new treatments and services outside the classical perimeter of actions. In this paper, we propose an Internet-of-People framework (Fig. 8) as a smart and digital corpus of innovative solutions. It includes connectedness, collective awareness, multiplexity, sharing and social environment to obtain changes in behaviours through a people-oriented network giving a personalised, participative and preventive service thanks to structured human-related data.

## Electronic supplementary material

Supplementary File
