# Supplementary material for: Quantifying the propagation of distress and mental disorders in social networks
Source: Sci Rep. 2018 Mar 22;8:5005. doi: 10.1038/s41598-018-23260-2 (PMC5864966; doi:10.1038/s41598-018-23260-2)
Supplement: Supplementary file 1 — Supplementary File [file 41598_2018_23260_MOESM1_ESM.pdf]

Supplementary File

Quantifying the propagation of distress and mental disorders in social networks

Marialisa Scatà[1,*], **Alessandro Di Stefano**[1], **Aurelio La Corte**[1] **and Pietro Liò**[2]

[1]University of Catania, Dipartimento di Ingegneria Elettrica, Elettronica e Informatica, Catania, 95125, Italy

[2]University of Cambridge, Computer Laboratory, Cambridge (UK), CB3OFD, UK

* corresponding author (lisa.scata@dieei.unict.it)

[+]these authors contributed equally to this work

Supplementary Figure S1. Overlapping awareness and centrality on weighted multiplex network. The color of the nodes represents the overlapping awareness A^{π} while the nodes' size corresponds to the awareness measure and the edges thick is the weight of [1]the multiplex network with overlapping awareness. On the left, we show the multiplex structure with overlapping awareness. On the right, we show the multiplex with overlapping awareness after having applied the rewiring based on the centrality measures of nodes and layers, choosing the links of the less central nodes in the less central layer. Figure shows how this heterogeneity, due to the simultaneous presence of the contagion phenomenon and other issues related to it, impacts on the structural parameters of the weighted multiplex network. This, in turns, acts on the coevolution of social contagion and awareness spreading, speeding up the latter process in comparison with the former. Rewiring of the multiplex network leads to a major heterogeneity of the multiplex network, which means an increased rate of awareness and subsequently a lower infection rate.

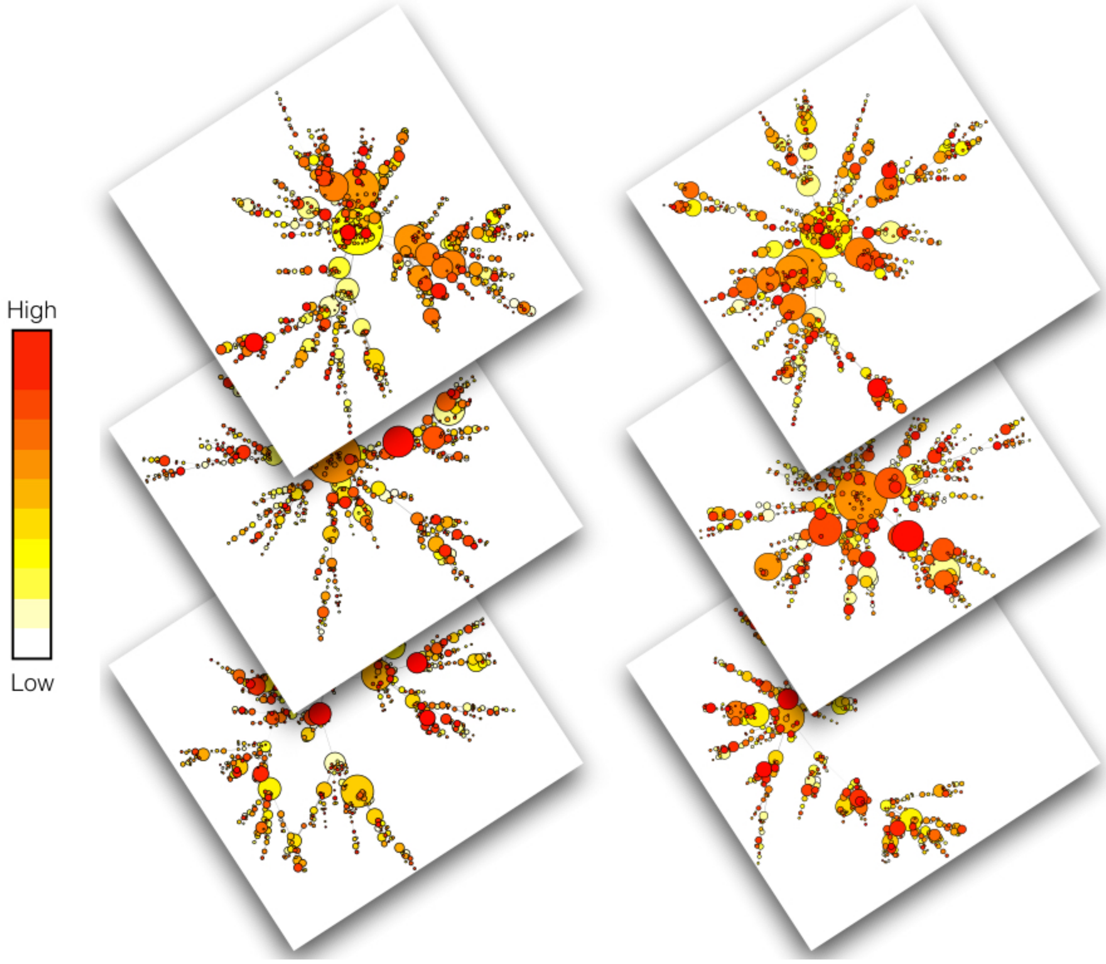

Supplementary Table S1. Data-driven approach: Awareness and Suicidal communication classes. We define the awareness score, based on three measures (measure for class, Google Trends and overlapping awareness). This is associated to each class referred to types of suicidal communication as in (41) with the corresponding keywords selected as representative for each class.

<table>
<tr><th colspan="2">Awareness Score</th><th>Timing</th><th>Suicidal Communication Classes</th><th>Associated Keyword</th></tr>
<tr><td>Awareness measure for classes</td><td></td><td rowspan="4">Pre-Event
(June 10, 2014 - August 10, 2014)

Suicide Event
Robin Williams' suicide*
August 11, 2014

Post-Event
(August 12, 2014 - October 10, 2014)</td><td rowspan="4">C1
C2
C3
C4
C5
C6
C7</td><td rowspan="4">'commit suicide'
'suicide support'
'i want to die'
'suicide prevention'
'suicide memorial'
'news on suicide'
none of the above</td></tr>
<tr><td>Google Trends measure</td><td></td></tr>
<tr><td rowspan="2">Overlapping Awareness measure</td><td>positively correlated case</td></tr>
<tr><td>anti-correlated case</td></tr>
</table>

* http://www.bbc.com/news/entertainment-arts-28765693

Supplementary Figure S2. Google Search Popularity of terms related to Suicide Contagion. We show scatter plots for Google search popularity of keywords related to the classes of suicidal communication types (41) over the period 2012-2017.

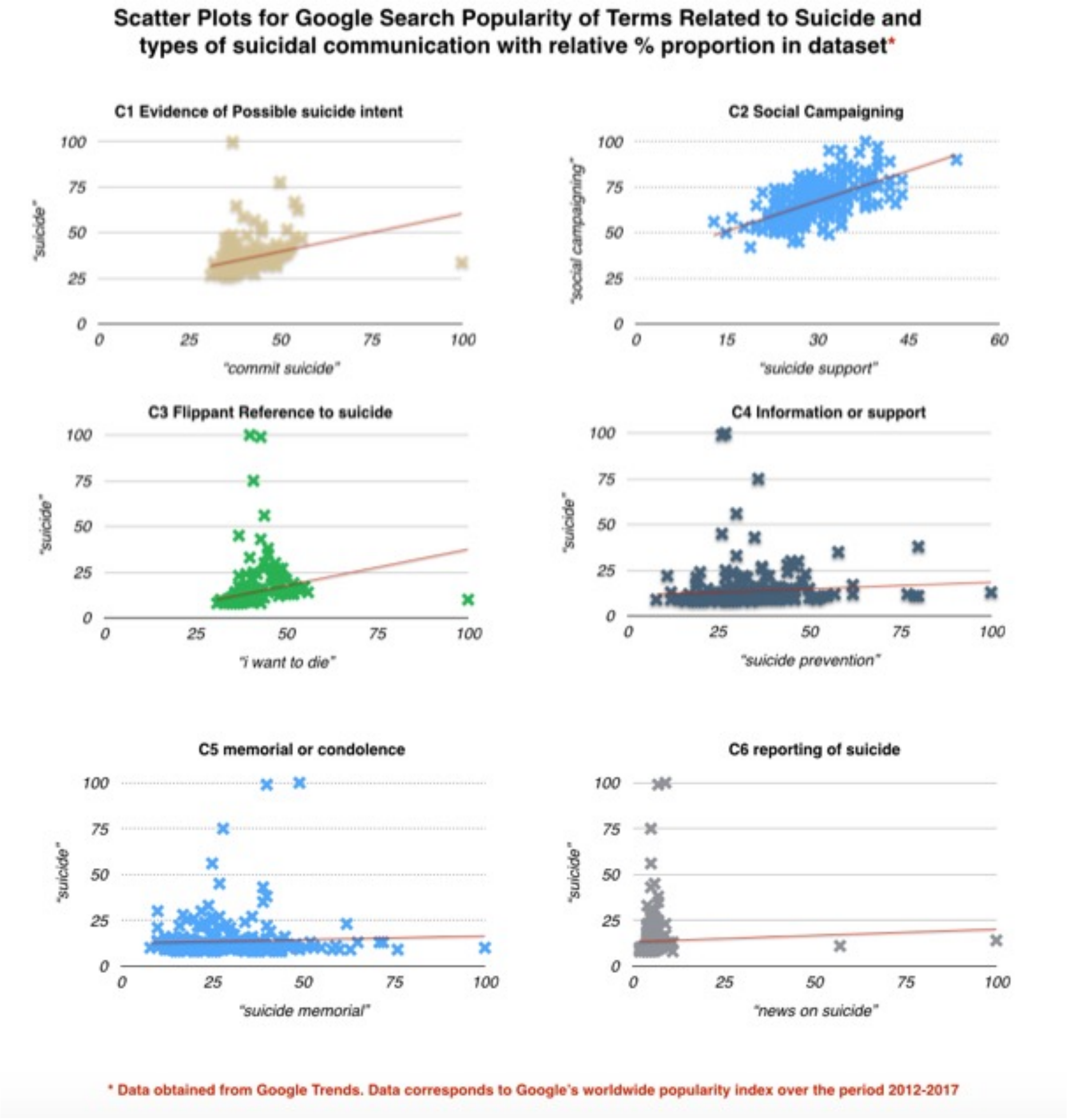

Supplementary Figure S3. Map of Suicide Rates and Google Search Popularity of Terms related to Suicide Contagion in 2015. Maps shows the Google Search Popularity of `Suicide', `Commit Suicide' and `Suicide Prevention', in comparison with Suicide Rates of the same countries (Graphics generated with Plotly, Plotly Technologies Inc. Collaborative data science. Montreal, QC, 2015, URL: **https://plot.ly**, Last Revised: February 10, 2017)

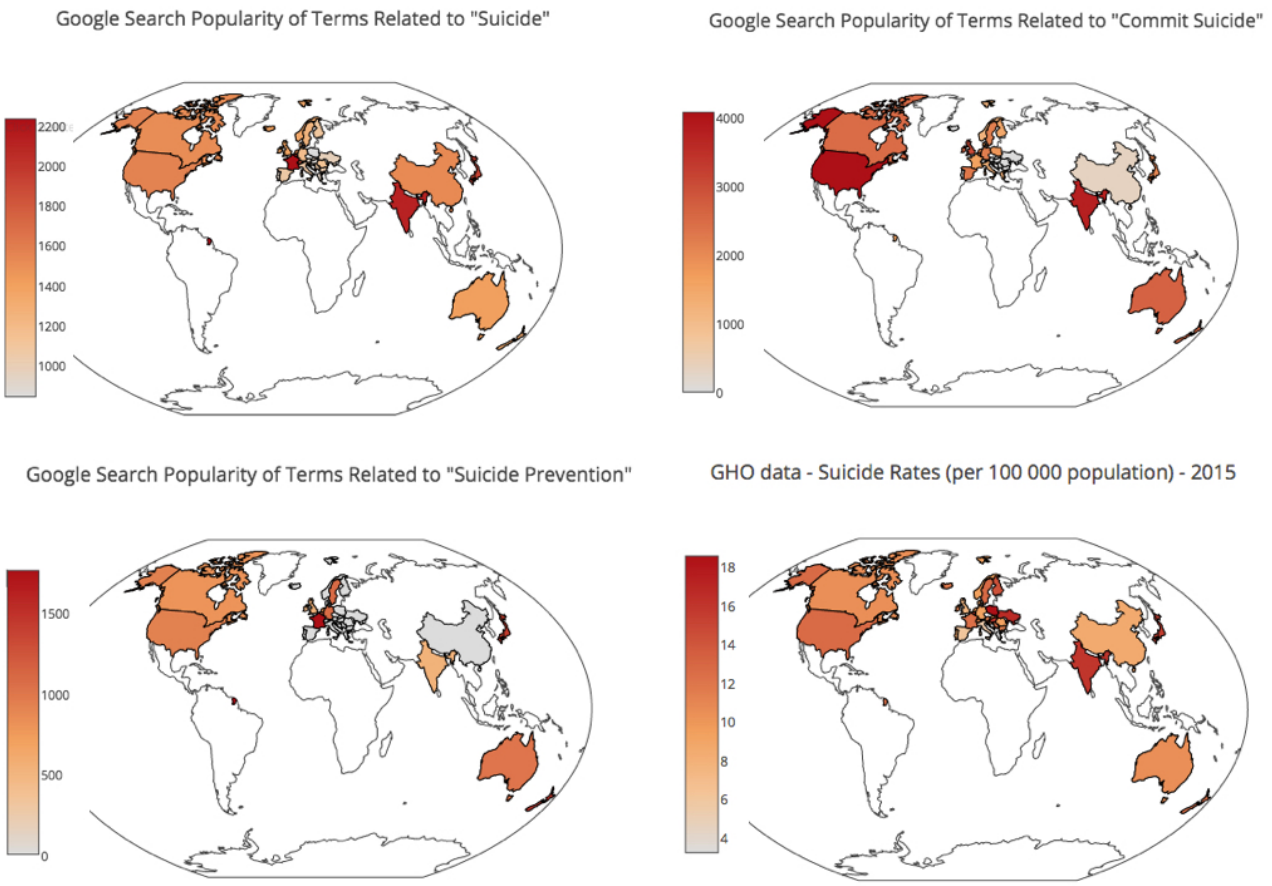

* Data obtained from Google Trends. Data corresponds to Google's worldwide popularity index over the year 2015
